# Telomere length in blood, buccal cells, and fibroblasts from patients with inherited bone marrow failure syndromes

**DOI:** 10.18632/aging.100235

**Published:** 2010-11-23

**Authors:** Shahinaz M. Gadalla, Richard Cawthon, Neelam Giri, Blanche P. Alter, Sharon A. Savage

**Affiliations:** ^1^ Clinical Genetics Branch, Division of Cancer Epidemiology and Genetics, National Cancer Institute, National Institutes of Health, Rockville, MD 20852, USA; ^2^ Cancer Prevention Fellowship Program, National Cancer Institute, Rockville, MD 20852, USA; ^3^ Department of Human Genetics, University of Utah. 15 N 2030 E, Room 2100, Salt Lake City, UT 84112, USA

**Keywords:** telomere, correlation, dyskeratosis congenita, bone marrow failure

## Abstract

Telomeres, the nucleotide repeats and protein complex at chromosome ends, are required for chromosomal stability and are important markers of aging. Patients with dyskeratosis congenita (DC), an inherited bone marrow failure syndrome (IBMFS), have mutations in telomere biology genes, and very short telomeres. There are limited data on intra-individual telomere length (TL) variability in DC and related disorders. We measured relative TL by quantitative-PCR in blood, buccal cells, and fibroblasts from 21 patients with an IBMFS (5 Diamond-Blackfan anemia, 6 DC, 6 Fanconi anemia, and 4 Shwachman-Diamond syndrome). As expected, TL in patients with DC was significantly (p<0.01) shorter in all tissues compared with other IBMFS. In all disorders combined, the median Q-PCR TL was longer in fibroblast and buccal cells than in blood (overall T/S ratio=1.42 and 1.16 vs. 1.05, p=0.001, 0.006, respectively). Although the absolute values varied, statistically significant intra-individual correlations in TL were present in IBMFS patients: blood and fibroblast (r=0.66, p=0.002), blood and buccal cells (r=0.74, p<0.0001), and fibroblast and buccal cells (r=0.65, p=0.004). These data suggest that relative TL is tissue-independent in DC and possibly in the other IBMFS.

## INTRODUCTION

Telomeres consist of long (TTAGGG)_n_ nucleotide repeats and an ordered protein complex at the ends of chromosomes that are essential to maintaining chromosomal integrity [1]. Telomere length (TL) shortens with each cell division and thus is an important marker of aging. It is also associated with the risk of several degenerative and age related diseases, including cancer [2,3]. Individuals with dyskeratosis congenita (DC), a cancer susceptibility and inherited bone marrow failure syndrome (IBMFS), have mutations in genes important in telomere biology, including *DKC1, TERC, TERT, TINF2, NHP2* and *NOP10* [4]. They also have very short telomeres for age compared with unaffected family members or age-matched controls [5]. Typically, DC is clinically defined by the triad of lacy, reticular skin pigmentation, nail dystrophy, and oral leukoplakia. Patients are at very high risk of bone marrow failure, cancer, pulmonary fibrosis, and other medical complications [4,6]. The presence of very short telomeres in leukocyte subsets as measured by auto-mated multicolor fluorescence *in situ* hybridization combined with flow-cytometry (flow-FISH) [7], is a useful tool for differentiating patients with DC from healthy individuals [8,9].

TL abnormalities have been reported in a few patients with other IBMFS, including Fanconi anemia (FA) [10,11], Shwachman Diamond syndrome (SDS) [12], and, to a lesser extent, in Diamond Blackfan anemia (DBA) [13], but the degree of telomere shortening is usually not as profound as in DC. When compared with DC, the short telomeres in patients with non-DC IBMFS are mainly observed in granulocytes [8], suggesting the presence of rapid granulocyte stem/progenitor cell turnover in response to bone marrow stress.

Data on the comparability between tissue TL, and correlations between TL measured in different tissues from the same individual are limited. Some studies, mostly in the elderly, suggest a possible variability in TL across tissues [14-17]. Studies of telomere length in cancer and other diseases have only evaluated either the abnormal tissue [18-20], peripheral blood [21-23], or buccal cells [24,25]. In one study, the average TL in five different tissue types, cerebral cortex, myocardium, liver, renal cortex and liver, was found to correlate within an individual [26].

**Table 1.** Demographic and Clinical Characteristics of Study participants Studies of TL in DC patients also showed short telomeres in cultured skin fibroblasts [27] and keratinocytes [28].

	Combined IBMFS N=21	By Disorder			
		DC N=6	FA N=6	DBA N=5	SDS N=4
N (%)					
Age at sample drawing (yrs)					
Median (range)^	21 (8-43)	16 (10-31)	27 (16-33)	30 (8-43)	12.5 (11-42)
Gender					
Male	15 (71.4%)	6 (100%)	3 (50%)	4 (80%)	2 (50%)
Female	6 (28.6%)	0	3 (50%)	1 (20%)	2 (50%)
Aplastic Anemia					
None	9 (43%)	1	4	3	1
Moderate	7 (33%)	4	0	0	3
Severe	5 (24%)	1	2	2*	0

^ The majority of samples for patients with IBMFS were collected on the same clinic day. The maximum time interval between sample collections in an individual patient was 1.8 years.

* Two patients with DBA had severe anemia requiring red blood cell transfusions for one patient, and prednisone in the other. Their white blood count and platelet counts were not affected.

Abbreviations: IBMFS, inherited bone marrow failure syndromes; DC, dyskeratosis congenita; FA, Fanconi Anemia; DBA, Diamond Blackfan Anemia; SDS, Shwachman Diamond Syndrome; N, number; Kb, kilobases

In order to better understand intra-individual TL variation in IBMFS, we compared TL, measured by Q-PCR, in DNA extracted from blood, buccal cells, and cultured fibroblasts within and between the four major IBMFS disorders, DC, FA, SDS, and DBA. We also assessed TL intra-individual correlations between tissues overall and within disorders.

## RESULTS

**Figure 1.**
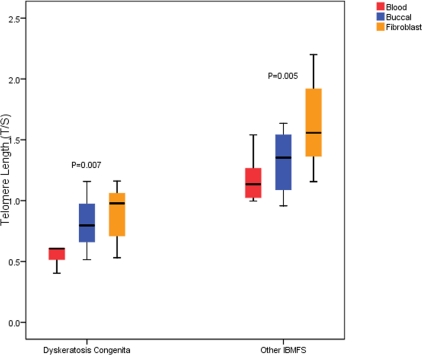
Comparison of Q-PCR blood, fibroblast and buccal cell DNA telomere length (TL) in DC patients, and patients with other IBMFS combined. Q-PCR TL (on the Y axis) is measured as a ratio of telomere copy number (T) to single copy gene (S) (T/S); p-values represent the global difference in tissues TL and obtained from Friedman test. Abbreviations: IBMFS, inherited bone marrow failure syndromes.

Table 1 summarizes the characteristics of the study participants. The ages of the patients ranged from 8 to 43 years (median=21), most were males (71.4%), and about one-fourth had severe aplastic anemia. As expected, telomeres were significantly shorter in patients with DC than in patients with other IBMFS in blood (median T/S, or relative TL=0.61 vs. 1.13, p=0.001), buccal cells (0.80 vs. 1.25, p=0.009), and fibroblasts (0.84 vs. 1.56, p=0.001) (Figure 1). In all IBMFS patients combined, median TL was longer in fibroblasts and buccal cells when compared with blood (1.42 and 1.16 vs. 1.05, p=0.001, 0.006, respectively) (Figure1). The same pattern was observed across each specific IBMFS (Table 2)

When evaluating the intra-individual correlations be-tween TL measurements in blood, buccal and fibroblast trios in all IBMFS patients, we observed significant correlations between blood and fibroblasts (r=0.66, p=0.002), blood and buccal cells (r=0.74, p<0.0001), and fibroblasts and buccal cells (r=0.65, p=0.004) measurements. Disease-specific analyses showed that this correlation was primarily derived from the strong correlation between TL in blood and buccal (r=0.90, p=0.04), buccal and fibroblast (r=0.90, p=0.04) in patients with DC, and TL in blood and fibroblast (r=1.0, p<0.0001) in patients with SDS. Likewise, we observed a positive, however not statistically significant, correlation between TL in buccal cells and blood in patients with FA (r=0.60, p=0.28) and DBA (r=0.70, p=0.19). A positive non-significant correlation was also observed between fibroblast and buccal TL in patients with DBA (r=0.70, p=0.19). Surprisingly, a strong negative correlation was present between fibroblast and buccal TL (r=-1.0, p<0.0001) in patients with FA (Table 3).

**Table 2.** Blood, buccal cells, and fibroblast Q-PCR telomere length in all participants combined and by disorder

Tissue type	Number	Median TL	Range	p-value*
**All patients with IBMFS**				
Blood	21	1.05	0.40-1.86	Reference
Buccal cells	19	1.16	0.51-6.44	0.006
Fibroblasts	20	1.42	0.47-2.20	0.001
**Dyskeratosis Congenita**				
Blood	6	0.61	0.40-0.99	Reference
Buccal cells	5	0.80	0.51-1.16	0.04
Fibroblasts	6	0.84	0.47-1.16	0.17
**Fanconi Anemia**				
Blood	6	1.13	0.79-1.35	Reference
Buccal cells	5	1.22	0.96-1.46	0.70
Fibroblasts	5	1.73	1.39-2.20	0.04
**Diamond-Blackfan Anemia**				
Blood	5	1.13	0.99-1.86	Reference
Buccal cells	5	1.52	1.03-2.23	0.08
Fibroblasts	5	1.60	1.15-1.69	0.14
**Shwachman-Diamond Syndrome**				
Blood	4	1.18	0.99-1.54	Reference
Buccal cells	3	1.39	1.04-6.44	0.11
Fibroblasts	4	1.48	1.18-2.17	0.07

* Wilcoxon signed rank test for paired samples

Abbreviations: TL, telomere length; IBMFS, inherited bone marrow failure syndromes

**Table 3.** Correlation coefficients (r) between telomere lengths measured by Q-PCR in blood, buccal cells and fibroblasts DNA.

Tissue type	Blood		Fibroblast		Buccal cells	
	N	r^^^	N	r^^^	N	r^^^
**All patients with IBMFS**						
Blood	21	1.00	20	0.66**	19	0.74**
Fibroblast	20	0.67**	20	1.00	18	0.65**
**Dyskeratosis Congenita**						
Blood	6	1.00	6	0.45	5	0.90*
Fibroblast	6	0.43	6	1.00	5	0.90*
**Fanconi anemia**						
Blood	6	1.00	5	-0.10	5	0.60
Fibroblast	5	-0.10	5	1.00	4	-1.00**
**Diamond-Blackfan Anemia**						
Blood	5	1.00	5	0.30	5	0.70
Fibroblast	5	0.30	5	1.00	5	0.70
**Shwachman-Diamond Syndrome**						
Blood	4	1.00	4	1.00**	4	0.50
Fibroblast	4	1.00**	4	1.00	4	0.50

^ Spearman rank correlation coefficient; *p ≤ 0.05; ** p ≤ 0.01

Abbreviations: IBMFS, inherited bone marrow failure syndromes

## DISCUSSION

Proper telomere length and structure are critical for chromosomal stability. Telomeres shorten with each cell division and are also susceptible to oxidative stress. TL is an important marker for aging, and appears to be associated with risk of several diseases, including cancer. Telomere biology disorders encompass a broad clinical spectrum, ranging from dyskeratosis congenita with its diagnostic triad and young age of onset, to isolated aplastic anemia or pulmonary fibrosis diagnosed later in life [29]. Telomeres have been reported to be shorter than normal in some patients with FA, SDS, and DBA, but in these cases they are not as abnormally short as in DC. In this study, we sought to understand the variability in telomere length within and between different tissues derived from patients with these IBMFS.

In all IBMFS patients combined, TL in DNA samples extracted from blood was significantly shorter than that extracted from fibroblasts or buccal cells; a pattern that was observed across disorders, but did not reach statistical significance in some of them. More than half (57%) of our patients had moderate to severe aplastic anemia and the remainder are at high risk of its development. It is possible that the shorter telomeres in patients' blood cells could be a result of higher telo-mere attrition due to the relatively rapid turnover of hematopoietic cells in response to bone marrow stress.

In DC, it is also due to their underlying defect in telomere biology. Our finding of longer telomeres in fibroblasts than in blood cells is in agreement with another report of TL in healthy centenarians and controls of different ages [16]. In support of this observation, a higher annual rate of telomere attrition has been reported in leukocytes (approximately 30bp/year) [16,30,31] than in fibroblasts (15bp/year) [32]. When comparing blood and buccal telomere length, our finding was discordant with the reported longer telomeres in leukocytes compared with buccal cells in patients with Alzheimer disease and their age-matched controls [17]; this difference could be due to differences in disease biology, different DNA extraction methods and/or limitations in sample size.

Although the absolute TL values varied between blood, buccal cells, and fibroblast DNA, we observed strong intra-individual correlations between the three tissues; buccal cells showed consistent correlation across IBMFS; however, this correlation did not reach statistical significance when the disorders were evaluated individually. This finding is in contrast to the observed lack of correlation between blood and buccal TL in healthy individuals and patients with Alzheimer's disease reported by another study [17]. Surprisingly, we observed an inverse correlation between buccal cell and fibroblast TL in patients with FA. This could theoretically be related to the high oral cancer susceptibility in FA patients. Of the 5 patients with FA included in this study, one had oral cancer 3 years prior to as well as 2 years after sample collection; a second patient with FA had oral cancer 5 years after sample collection. Larger sample sizes are required to better understand these findings. Our observed strong correlation between blood and fibroblast TL in patients with SDS is an interesting finding that needs further evaluation.

TL correlations in different tissues have been observed in several other studies. Specifically, between fibroblasts and leukocytes (r^2^=0.80, p=0.0006)[15], bone marrow and skin (r^2^ = 0.77), vocal fold and blood (r^2^ = 0.84), skin and vocal folds (r^2^=0.62)[33], epidermal and lingual epithelium (r=0.84, p<0.0001) [34], and each pair of the following tissues: cerebral cortex, myocardium, liver, and renal cortex [26]. In our study, we observed a strong correlation profile between evaluated DNA samples in patients with DC, where heritable telomere dysfunction is present, particularly between blood and buccal cells (r=0.90) and buccal cells and fibroblasts (r=0.90). This suggests that short telomeres in DC are not limited to blood or fibroblasts, observations that have been previously reported [27], but are also present in buccal cells. This finding also suggests that the heritability of telomere length in DC is tissue independent.

In conclusion, overall Q-PCR TL in patients with IBMFS was correlated between blood, buccal cells and fibroblasts, but there was some variability within different IBMFS. This small pilot study suggests that blood or tissue Q-PCR TL could theoretically be useful as a first screen for individuals with a suspected telomere biology disorder. However, much larger studies are needed to evaluate the reliability and validity of this test in comparison to the standard diagnostic method of flow-FISH.

## METHODS

### Ethical statement

This investigation was conducted in accordance with the ethical standards and according to the Declaration of Helsinki and the national and international guidelines and has been approved by the National Cancer Institute's (NCI) institutional review board.

### Study participants

The study included 21 patients with IBMFS; 5 Diamond Blackfan anemia (DBA), 6 Dyskeratosis Congenita (DC), 6 Fanconi anemia (FA), and 4 Shwachman-Diamond syndrome (SDS) who were enrolled in the NCI study entitled “Etiologic Investigation of Cancer Susceptibility in Inherited Bone Marrow Failure Syndromes” and provided blood, buccal cells, and fibroblast samples. Study details are available elsewhere [35]. Briefly, evaluation of patients described in this cohort includes systematic review of medical records, careful physical examination, complete blood counts, bone marrow studies, skin biopsy, buccal cell collection, and other appropriate diagnostic tests.

### DNA extraction and telomere length measurements

Genomic DNA from blood cells was extracted from either whole blood (4 samples; 3 DBA and 1 DC) or the white blood cell pellet remaining after density gradient separation (17 samples, primarily granulocytes) by manual Gentra Puregene procedure (Qiagen Inc., Valencia, CA). Skin fibroblasts were cultured from punch biopsies and stored at -80°C. Buccal cells were collected using Scope mouthwash. DNA was isolated from fibroblasts and buccal cells by phenol-chloroform extraction.

TL was measured using the Quantitative- Polymerase Chain Reaction (Q-PCR) method previously described [36]. Briefly, the relative TL was estimated from the ratio of the telomere (T) repeat copy number to a single gene (human β-globin gene) (S) copy number (T/S ratio) for each sample using standard curves. For quality control, all Q-PCR TL measurements were done in triplicates. The average coefficients of variation (CV) between triplets for blood, buccal and fibroblast samples were 6.4%, 7.3%, and 7.9%, respectively.

### Statistical analysis

We used Wilcoxon signed-rank test to compare the median ranks of TL within each disorder (buccal, and fibroblast *vs.* blood), and Mann-Whitney test to compare tissue-specific TL between DC and other IBMFS. Spearman rank correlation coefficient (R) was used to evaluate the intra-individual correlations between Q-PCR TL in blood-fibroblast-buccal cell trios. Analyses were done using SPSS.17 statistical software.
